# Stroke units in Nigeria: a report from a nationwide organizational cross-sectional survey

**DOI:** 10.11604/pamj.2022.42.140.35086

**Published:** 2022-06-22

**Authors:** Babawale Arabambi, Olajumoke Oshinaike, Shamsideen Abayomi Ogun, Chukwuemeka Eze, Abiodun Hamzat Bello, Steven Igetei, Yakub Yusuf, Rashidat Amoke Olanigan, Sikirat Yetunde Ashiru

**Affiliations:** 1 School of Population and Public Health, The University of British Columbia, Vancouver, Canada,; 2 Department of Medicine, Neurology Unit, Lagos State University Teaching Hospital, Lagos, Nigeria,; 3 Department of Medicine, Lagos State University College of Medicine, Lagos, Nigeria,; 4 Department of Medicine, Alex Ekwueme Federal University Teaching Hospital, Abakaliki, Nigeria,; 5 Department of Medicine, University of Ilorin Teaching Hospital, Ilorin, Nigeria,; 6 Department of Internal Medicine, University of Benin Teaching Hospital, Benin City, Nigeria,; 7 Department of Medicine, Federal Medical Center, Asaba, Nigeria

**Keywords:** Stroke, hospital units, tertiary prevention, Nigeria, acute stroke care

## Abstract

**Introduction:**

stroke is one of the leading causes of death and disability in Nigeria. Stroke unit care is crucial for reducing mortality and morbidity in stroke. This study describes the stroke units' structure, organization, and care process in Nigerian tertiary hospitals.

**Methods:**

this study is a cross-sectional descriptive organizational survey-based study using an online structured questionnaire to collect information on the stroke units.

**Results:**

five (8.6%) out of 58 hospitals had a stroke unit. The number of beds ranged between 10 and 27 with the coverage of hospital stroke patients ranging from 24% to 100%. All the centers had a multidisciplinary team for their unit. The basic required investigations like computerized tomography and electrocardiography were available in the centers. Thrombolytic therapy coverage was suboptimal in all the centers due to prolonged onset-to-arrival times and inaccessibility of thrombolytic medications.

**Conclusion:**

there has been some progress in stroke unit availability since the country´s first stroke unit was established over a decade ago. However, there is still the need to create more stroke units in Nigeria and improve reperfusion therapy coverage.

## Introduction

Globally, stroke remains a leading cause of death and disability, with more than 10 million new stroke cases reported in 2019 alone, adding up to over 100 million people living with stroke by the end of that year [1]. In Nigeria, a country with over 200 million people [2], stroke was second to ischemic heart diseases as a cause of death and disability from a non-communicable disease according to the 2019 data [3,4]. Hospital-based studies in Nigeria between 2004 and 2021 reported 30-day case fatality rates ranging from 21.2% to 40% [5-9]. Proper acute stroke care is critical to survival and achieving favorable functional outcomes in stroke patients. In fact, the care of stroke patients in a stroke unit is a cornerstone of tertiary prevention. Management of stroke patients in a dedicated stroke unit has been shown to improve the chances of being less dependent in the long term and reduce the risk of death and institutionalization [10]. Indeed, stroke care guidelines recommend that stroke patients be treated acutely in an organized setting provided by a dedicated stroke unit [11].

A stroke unit is a special setting for managing stroke patients, with crucial components including a dedicated ward, a stroke care team that spans various disciplines, and a standard stroke care guideline. Members of the stroke team include neurologists, neurosurgeons, stroke nurses, physical and occupational therapists, speech therapists, dieticians, medical social workers, and other affiliated health professionals. The team works together harmoniously to deliver acute stroke care to the patients [10,12]. The World Stroke Organization (WSO) global stroke guidelines and quality action plan recommend that patients with acute stroke or transient ischemic attack be managed in a stroke unit with at least a stroke care physician, a nurse, and a rehabilitation specialist [13]. Essential services provided by an acute stroke unit should include neurological evaluation and diagnosis of stroke, including neuroimaging (CT scan), electrocardiography (ECG), reperfusion therapy (thrombosis), supportive treatment, monitoring, secondary prevention, and early intensive rehabilitation [13,14]. The Nigerian Stroke Organization (NSO) also recommended that “all stroke patients should be managed in a stroke unit” (Class I recommendation, Level A Evidence) [15]. A global survey reported that only 18% of low-income countries had stroke units, compared to 91% of high-income countries [16]. The first stroke unit in Nigeria was established in 2010 at the University of Benin Teaching Hospital [17]. In 2012, a literature review found that most stroke patients in Nigeria who presented at the hospital were treated in the general medical wards [18]. Given the importance of stroke unit care to the prognosis of the disease in stroke patients, it is necessary to describe the current state of stroke units in the country, focusing on availability, capacity, and capability. This could help support the push for advocacy to establish more stroke units, multi center stroke research collaborations, and standardize stroke care across the centers. This study aimed to describe the available stroke units in Nigerian tertiary hospitals, examining their structure, organization, and care process.

## Methods

**Study design:** this cross-sectional descriptive organizational survey-based study was reported using the “Consensus-based checklist for reporting of survey studies (CROSS)” guidelines [19].

**Setting:** this study was conducted in Nigeria, one of the most densely populated countries in sub-Saharan Africa [2]. The country is divided into six geopolitical zones - North-East (NE), North-Central (NC), North-West (NW), South-East (SE), South-South (SS), and South-West (SW). Nigeria has 36 states in addition to the Federal Capital Territory (FCT), Abuja [20]. Each Nigerian state has at least one tertiary hospital that provides care to patients with neurological disorders, including stroke.

**Participants:** the study population consisted of teaching hospitals (TH) and federal medical centers (FMC) in Nigeria. These hospitals are classified as tertiary centers because of their patient load and availability of specialist care. The THs are affiliated with different universities and provide medical students and residents training. Ownership and management of the centers were either by the federal government, state governments, or private institutions. The federal government manages the FMCs, typically situated in states without a TH. A total of 58 centers, including 38 THs and 20 FMCs, were identified as potentially eligible for the survey [21,22]. The inclusion of a center was based on the availability of a dedicated stroke unit.

**Variables:** the main outcomes were latent variables that described stroke unit structure and organization, stroke unit care process, discharge and follow-up, and data management in a stroke registry. Since this study was a descriptive rather than analytical study, predictors or confounders were not relevant to the context.

**Data sources and measurement:** the data collection instrument was a structured questionnaire with five sections. The first and second sections collected information on center classification and availability of a neurology unit and a stroke unit. The third section examined the stroke unit structure and organization with questions on capacity, staffing, patient load, criteria for admission, multidisciplinary meetings, and training programs. The fourth section focused on the unit's care process, including treatment guidelines, pre-admission procedures, available investigations, assessment scales, nursing and physiotherapy assessment, supportive care equipment, thrombolytic therapy, and mechanical thrombectomy. The final section elicited the discharge and follow-up plans and data management in a stroke registry. The questionnaire was formulated via consensus after discussion between the authors BA, SAO, and OO. Validation rules were attached to all the questions, and the authors pretested the instrument before deployment. The questionnaire (“A snapshot of stroke units in Nigeria”) was shared as a Google forms survey and administered between March 3^rd^ and 16^th^, 2022. The forms were shared with neurologists or neurology residents in centers with stroke units, who were actively involved in providing care to admitted stroke patients.

**Statistical methods:** data were qualitatively described and presented in tables. Selection of participating hospitals was summarized using a flowchart. Data cleaning, conversion, and summaries of frequencies and range were done using R studio [23].

## Results

**Figure 1:**
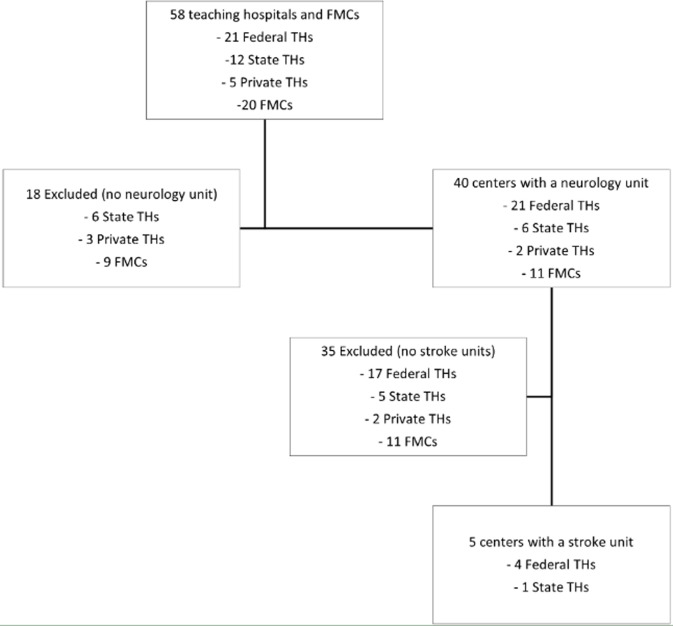
selection of eligible stroke units

Out of 58 hospitals, only five centers (8.6%) had a dedicated stroke unit (Figure 1). Of the five centers included in the study, two were in South-West Nigeria, one each in the country's south-south, south-east, and north-central regions. Four of them were federal THs, while the last one was a state TH.

**Stroke unit structure and organization:** the first center was established in 2010, while the most recent unit started in 2021. Most centers had ten beds in the stroke ward, with the biggest center operating with 27 beds. In each shift, the nurses per bed ranged from one nurse per seven beds to one nurse per two beds. The stroke units admitted 24% to 100% of all stroke patients in these centers six months before the survey. All the centers had neurosurgeons (NS), physiotherapists (PTs), radiologists, and medical social workers (MSWs), while most of the centers had speech and language therapists (SLTs), psychiatrists, and nutritionists. However, only two centers had occupational therapists (OTs). Typical exclusion criteria for admission into the stroke wards included stroke duration greater than seven days and severe strokes requiring intensive care. Most centers had multidisciplinary team meetings and educational programs for their staff. Other details are shown in Table 1.

**Table 1:** stroke unit structure and organization

Center name	AEFUTHA	UITH	UBTH	LASUTH	LUTH
Year established	2021	2012	2010	2019	2017
Number of beds	10	14	27	10	10
Neurologists to bed ratio	1 to 5	1 to 7	1 to 7	1 to 3	1 to 3
Nurses to bed ratio per shift	1 to 2	1 to 7	1 to 7	1 to 3	1 to 3
Dedicated stroke unit doctors round the clock?	Yes	No	Yes	Yes	Yes
Percentage of hospital stroke patients admitted in stroke ward	50%	50%	100%	24%	71%
Admissions per month in the stroke ward	20	15	20	50	25
Available specialists (count)	NS (2) PT (10) Psychiatrists (8) MSW (5) Nutritionists (10)	NS (3) PT (8) Psychiatrists (10) SLT (1) MSW (3) Nutritionists (3)	NS (4) PT (4) Psychiatrists (5) OT (3) MSW (1) Nutritionists (1)	NS (3) PT (6) Psychiatrists (5) OT (1) SLT (1) MSW (3) Nutritionists (2)	NS (3) PT (4) Psychiatrists (5) MSW (2) Nutritionists (1)
Multidisciplinary meetings frequency	Less than once a week	Rarely	Rarely	Once a week	Less than once a week
Multidisciplinary meetings with patient relatives	No	No	No	No	Yes
Training programs for staff	Yes	Yes	No	Yes	Yes

MSW: medical social workers; NS: neurosurgeons; OT: occupational therapists; PT: physiotherapists; SLT: speech and language therapists

**Stroke unit care process:** all the centers had definite, accessible guidelines for managing patients admitted into the stroke unit. Patients admitted to the units had detailed neurological history and examination taken, were assessed early by PT/OT, had swallow tests, strict input-output charting, and closely monitored vital signs. All the centers had at least a brain computerized tomography (CT) available for their stroke patients, though the CT suites were not domiciled in the stroke unit. Further details of the care processes are shown in Table 2. Centers also had facilities for viral studies like SARS-CoV-2 and HIV and had access to typical investigations for evaluation of stroke in young people. Only two centers had given thrombolytic therapy to their stroke patients. The lack of thrombosis was due to affordability, unavailability of medications, delayed onset-to-arrival time and delayed door-to-needle time. None of the centers were capable of a mechanical thrombectomy procedure.

**Table 2:** stroke unit care process

Center name	AEFUTHA	UITH	UBTH	LASUTH	LUTH
Available investigations	CT scan	CT scan	CT scan	CT scan	CT scan
	ECG	ECG	ECG	MRI	MRI
	Biochemistry	Biochemistry	Biochemistry	ECG Biochemistry	ECG Biochemistry
	Hematology	Hematology	Hematology	Hematology	
	Chest X-ray	Chest X-ray	Chest X-ray	Chest X-ray	Chest X-ray
	Echocardiography	Echocardiography	Echocardiography	Echocardiography	Echocardiography
		Carotid doppler		Carotid doppler	Carotid Doppler
Routine stroke assessment scales	mRS	mRS	CNS	mRS	mRS
		NIHSS		NIHSS	NIHSS
		ICH score		ICH score	
Available equipment/facilities in the unit	Intensive care Continuous cardiac monitoring	Continuous cardiac monitoring	Supplemental oxygen	Continuous cardiac monitoring	Continuous cardiac monitoring
	High-dependency beds			High-dependency beds	
	Supplemental oxygen	Supplemental oxygen	Supplemental oxygen	Supplemental oxygen	Supplemental oxygen
	Pneumatic compression device	Pneumatic compression device		Pneumatic compression device	Pneumatic compression device
	Anti-pressure sore mattress	Anti-pressure sore mattress	Anti-pressure sore mattress	Anti-pressure sore mattress	Anti-pressure sore mattress
	Defibrillators		Defibrillators		Defibrillators
Past use of thrombolytic therapy (count)	No	No	Yes (1)	Yes (4)	No
Mechanical thrombectomy capability	No	No	No	No	No

CNS: Canadian neurological scale; ECG: electrocardiography; ICH: intracerebral hemorrhage; MRI: magnetic resonance imaging; mRS: modified Rankin scale; NIHSS: National Institute of health stroke scale

**Stroke unit discharge/follow-up and data management:** the average length of stay in the stroke units ranged from 7 to 20 days, with the usual discharge destination being the neurology clinic. Three centers had their local stroke registry where they input the data for admitted patients. Two of the centers (LASUTH and LUTH) participated in an international stroke registry.

## Discussion

This study provides a snapshot of stroke units in Nigeria. The mode of collection of information may be associated with some accuracy issues. Still, the breadth of information given is a good starting point for describing what is available for this vital aspect of stroke care. Stroke unit coverage in Nigeria is still suboptimal, with only a few centers having stroke units. The units in most of these centers are not large enough to provide care for all admitted acute stroke patients. Besides guidelines that recommend stroke unit care for better patient outcomes [10,11], several other studies have supported this recommendation in real-life settings across different geographical contexts [24-30]. A previous study in Australia concluded that any hospital that admits more than 100 stroke patients in a year should have a stroke unit [31]. Although we do not have the data on annual stroke admissions in Nigerian tertiary hospitals from our study, we can extrapolate from the five included centers that most of the other centers would cross the threshold for the need for a stroke center. None of the FMCs or private THs had a stroke unit. While the reason for this skewness is unclear, funding and staffing deficiencies may have a role to play. The hindrances to establishing stroke units in Nigerian tertiary hospitals are multifaceted. Implementation and maintenance of the unit are capital-intensive. Indeed, neuroimaging equipment, continuous cardiac monitoring devices, and well-trained staff are essential for the seamless operation of a stroke ward [32]. Resolving these challenges requires advocacy at the government level for the establishment of more stroke units [33]. Furthermore, collaboration with centers already providing standard stroke care for training, telemedicine, and research purposes is beneficial [32].

The availability of nurses in the stroke ward is directly associated with early stroke outcomes, with centers with a higher nurse-to-bed ratio performing better than those with a lower ratio [34,35]. Paley *et al*. suggested three trained nurses per 10 beds [34] for optimal outcomes. Some of the centers in these studies would benefit from increasing the number of nurses in their units. Multidisciplinary coverage was adequate in the centers; however, some centers did not have interdisciplinary team meetings with their patients and relatives. These meetings are essential because they provide an opportunity to harmonize management decisions, respond to patient concerns, allay their fears, and plan long-term rehabilitation and follow-up. The hospital length of stay of 7 to 20 days reported in this survey is comparable to reports of 10 days on average from previous studies in sub-Saharan African countries [36,37].

According to a survey of stroke experts, the minimum investigations required for stroke unit care were brain CT (prioritized for stroke patients), carotid Doppler, and ECG [38]. A previous study of stroke patients in a Cameroonian medical unit confirmed the importance of brain CT in mortality reduction in a resource-constrained setting [39]. Recent studies have suggested an increase in the prevalence of extracranial carotid atherosclerosis in Africans [40,41]. Additionally, carotid artery disease has been shown to increase the risk of stroke independently in Nigeria [42]. This trend suggests that a carotid Doppler may be necessary for all admitted stroke patients in Nigeria. The use of thrombolytic therapy remains a significant challenge. Inaccessibility of thrombolytic treatment has also been reported in other developing countries [33,36]. Known reasons for lack of thrombosis include cost [33] and delayed presentations, with prolonged onset-to-arrival and door-to-needle time [43]. For context, most health services in Nigeria are paid for out-of-pocket, with close to 97% of the population without any health insurance coverage [44]. While the minimum wage in Nigeria is 30,000 Naira (equivalent to 70 US dollars), the cost for a complete infusion of intravenous Alteplase, according to “drugstore.ng,” is over a million Naira (more than 2,500 US dollars). Therefore, thrombolytic therapy is prohibitively expensive and is not routinely stocked by pharmaceutical outlets. Furthermore, previous studies in Nigeria have identified significant delays in presenting to the hospital, with less than 10% of patients with ischemic stroke getting admitted within the thrombolytic window [6,45]. Reasons for delayed onset-to-arrival time, as identified from previous studies, include a lack of awareness of symptoms and the belief that stroke is a “spiritual attack” that is better treated using spiritual and herbal remedies [46,47]. Overall, while there have been more stroke units established since the first one in 2010, Nigeria still needs to improve tertiary stroke prevention by adding more stroke units to reduce the impact of stroke on mortality and disability. The loss of life years would affect not only the patient but also their caregivers and dependents. Additionally, existing units should be improved in terms of the number of nurses and the provision of thrombolytic therapy, with a plan to start mechanical thrombectomy. At the level of the government, advocacy for the establishment of more stroke units and subsidization of thrombolytic treatment will be beneficial in the long term. Potential challenges to establishing more stroke units and improving existing ones include cost, human resources, and incompatibility with existing systems. However, the evidence is unequivocal regarding the benefits of these units outweighing the costs.

## Conclusion

Setting up more and improving existing stroke units in the various centers in Nigeria should be advocated and encouraged to enhance tertiary prevention, thus reducing disability and mortality, and improving stroke outcomes.

### What is known about this topic

- *Stroke unit treatment are critical to improving stroke outcome;*
- *Stroke is one of the leading causes of mortality and morbidity in Nigeria*.

### What this study adds

- *Documents the status of stroke units in Nigeria regarding availability and capacity;*
- *Emphasizes the need to improve on the existing stroke units in addition to establishing more units*.
